# Supplementary material for: High‐resolution serum proteome trajectories in COVID‐19 reveal patient‐specific seroconversion
Source: EMBO Mol Med. 2021 Jul 7;13(8):e14167. doi: 10.15252/emmm.202114167 (PMC8687121; doi:10.15252/emmm.202114167)
Supplement: Supplementary file 2 — Appendix [file EMMM-13-e14167-s009.pdf]

Appendix

Appendix Fig S1 - Proteome correlation of COVID-19 patients

Appendix Fig S2 - Physiological process centric longitudinal protein trajectories

Appendix Fig S3 - Time-resolved SARS-CoV-2 antibody immunoassay responses of COVID-19 patients

Appendix Fig S4 - Proteins significantly correlating to the EUR S-IgG assay for patient 15

Appendix Fig S5 - Proteins correlating to different SARS-CoV-2 antibody assays

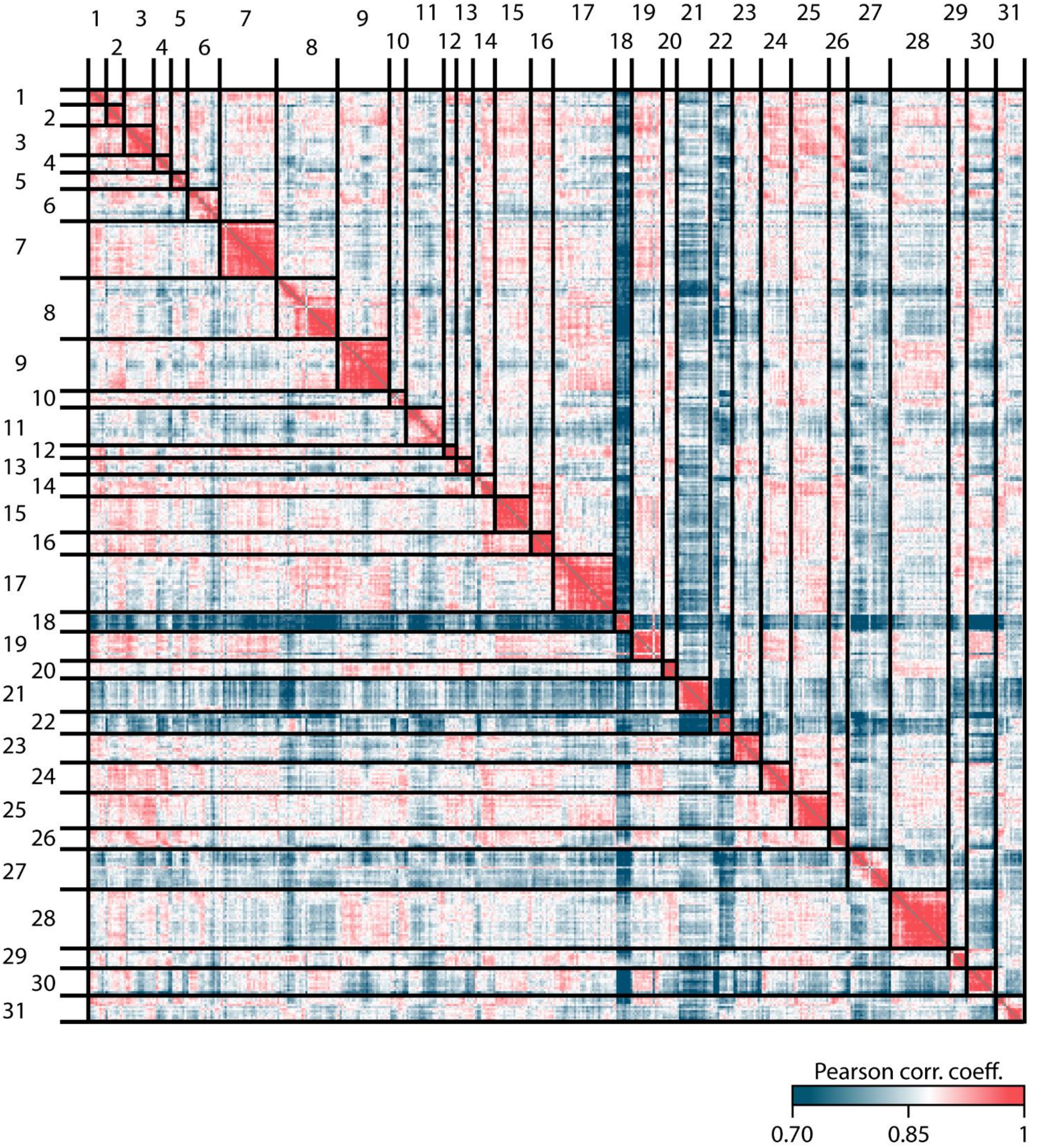

Appendix Fig S1 - Proteome correlation of COVID-19 patients

Individuals are numbered from 1 to 31 and samples are ordered according to the day of sampling.

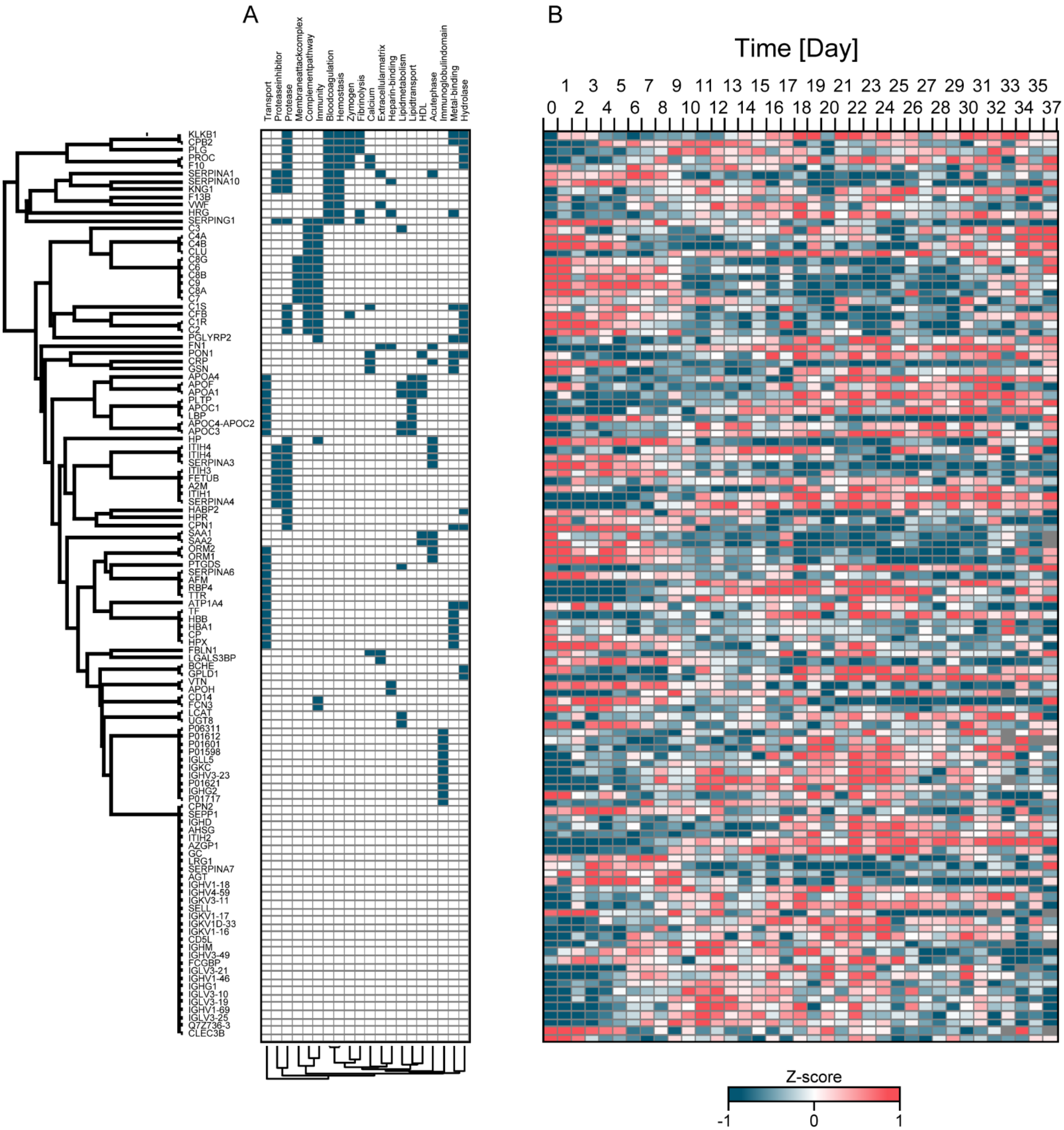

Appendix Fig S2 - Physiological process centric longitudinal protein trajectories.

A. Main keywords associated with regulated proteins are highlighted and hierarchical clustering sorts proteins into similar annotated groups.

B. Longitudinal protein trajectories in COVID-19 over a sampling time of up to 37 days represented as a heatmap and clustered as in (A) according to the keyword clustering.

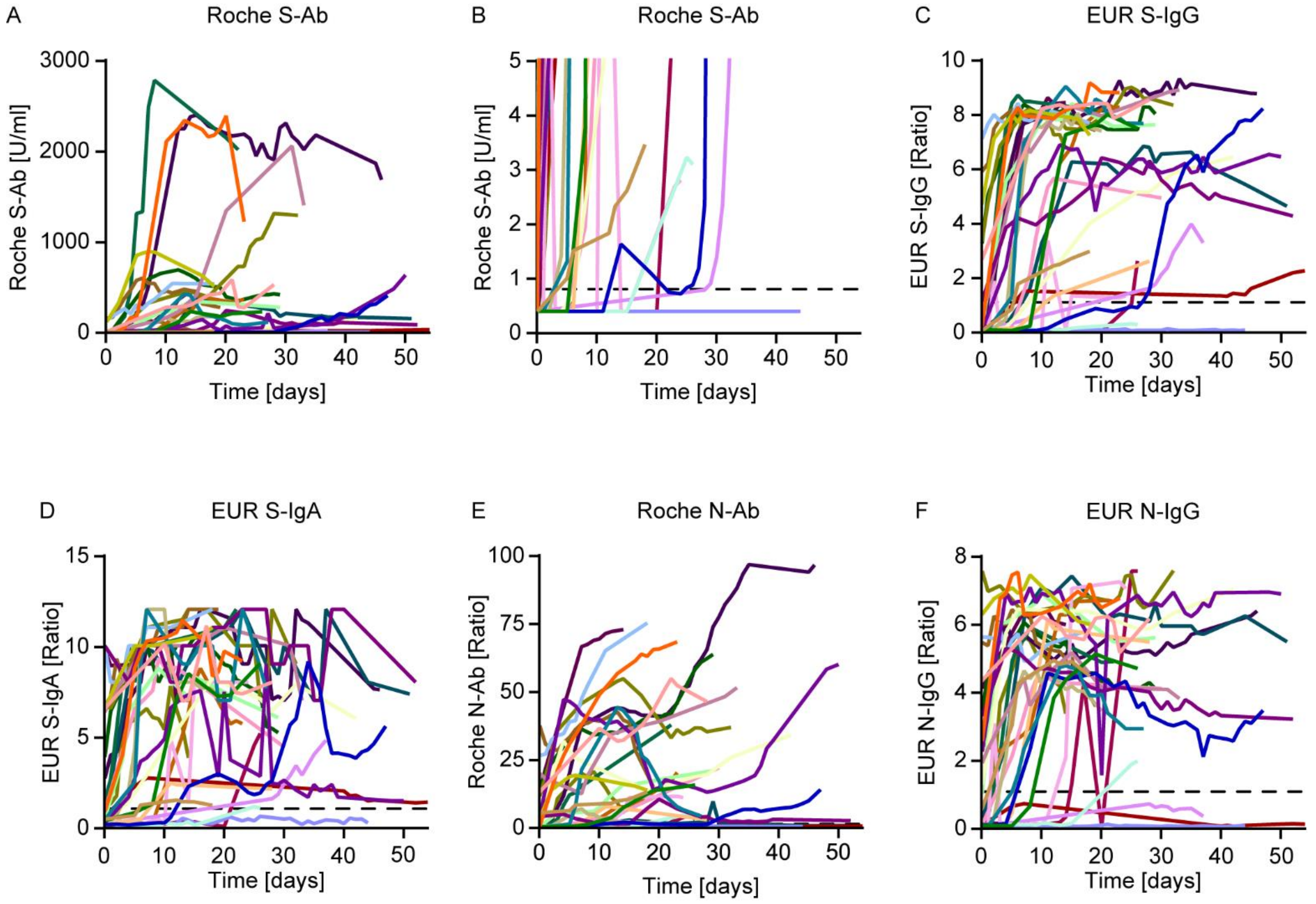

Appendix Fig S3 - Time-resolved SARS-CoV-2 antibody immunoassay responses of COVID-19 patients

A. Trajectories of the Roche S-Ab.

B. Zoom-in of the Roche S-Ab response from (A). Dashed line indicates a positive test result.

C-F. Longitudinal trajectories of the EUR S-IgG, EUR S-IgA, Roche N-Ab, and EUR N-IgG test, respectively.

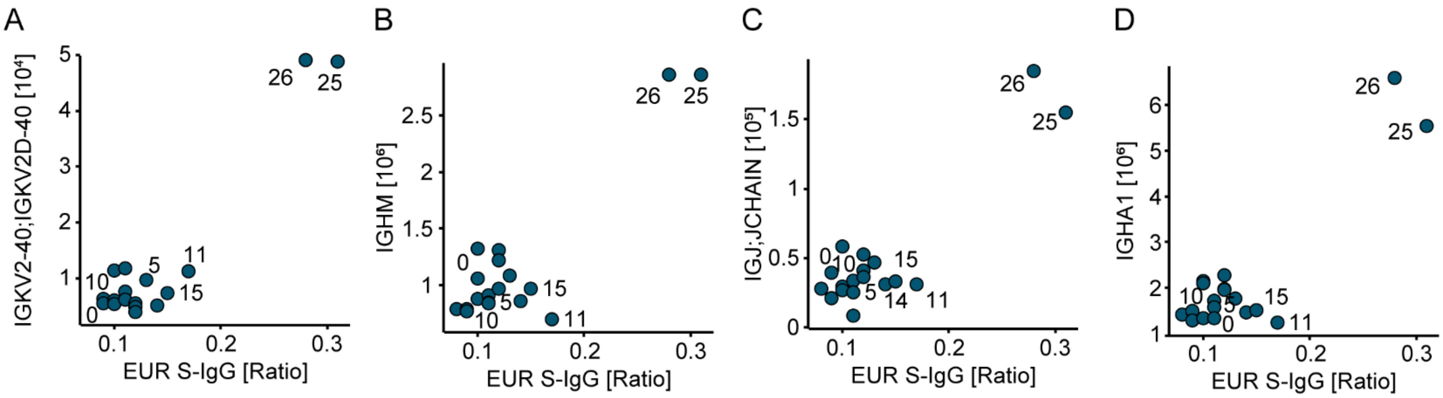

Appendix Fig S4 - Proteins significantly correlating to the EUR S-IgG assay for patient 15

A-D. Examples of correlations of four different immunoglobulin regions measured with the EUR S-IgG test within one patient. Labels indicate the day of sampling. The data points of early sampling days are clustered near the origin, in contrast to later sample dates where both protein abundance values and EUR S-IgG values are increased, consistent with seroconversion.

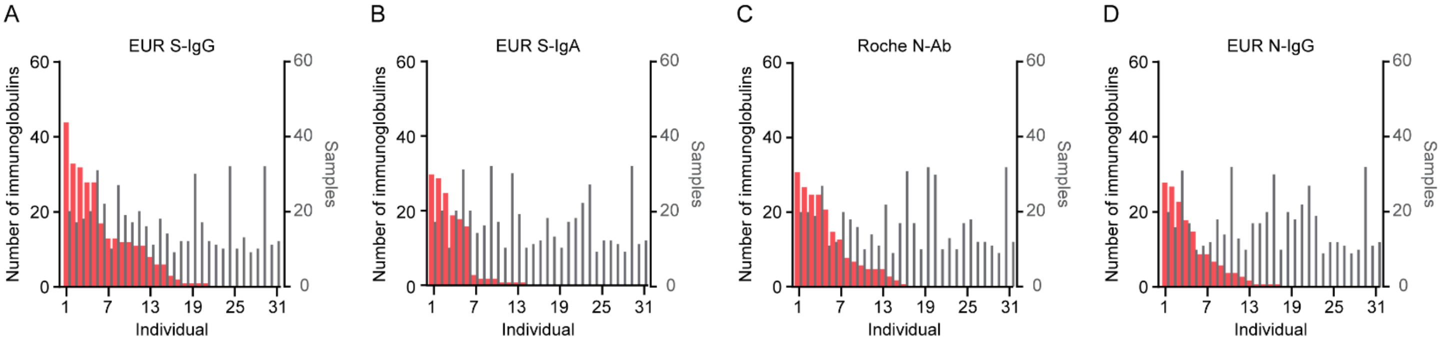

Appendix Fig S5 - Proteins correlating to different SARS-CoV-2 antibody assays

A-D. Number of immunoglobulins significantly correlating in the indicated individuals to EUR S-IgG (A), EUR S-IgA (B), Roche N-Ab (C), and EUR N-IgG test (D). Right y-axis indicates the number of samples for each individual.
