# Supplementary material for: High‐resolution serum proteome trajectories in COVID‐19 reveal patient‐specific seroconversion
Source: EMBO Mol Med. 2021 Jul 7;13(8):e14167. doi: 10.15252/emmm.202114167 (PMC8687121; doi:10.15252/emmm.202114167)
Supplement: Supplementary file 3 — Expanded View Figures PDF [file EMMM-13-e14167-s004.pdf]

Expanded View Figures

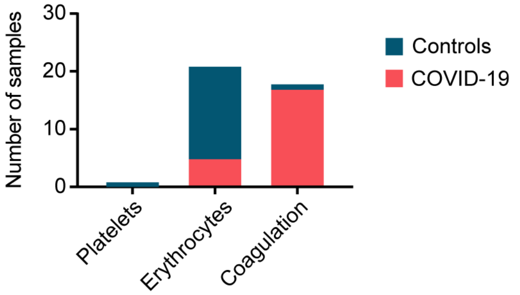

Figure EV1. Distribution of samples with poor quality according to platelet contamination, erythrocyte contamination, and coagulation in COVID-19 positive and negative patients.

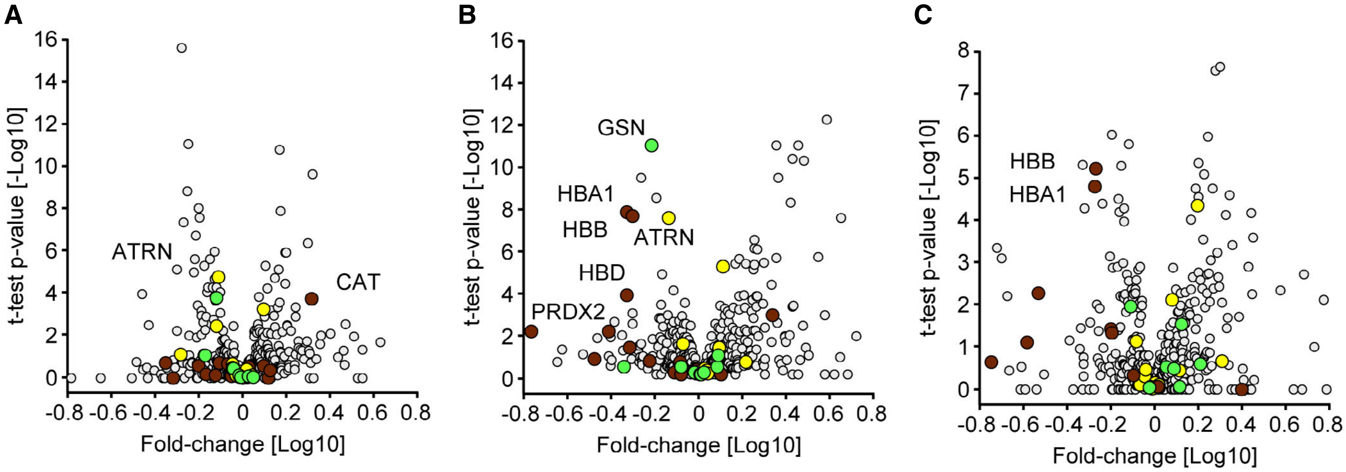

Figure EV2. Quality marker distribution in Volcano plots.

A Comparison of the serum proteomes of 31 COVID-19 patients on the first day of sampling and 262 PCR-negative controls according to Fig 2A. Erythrocyte markers are highlighted in dark red. Platelet markers are highlighted in green. Coagulation panel markers are highlighted in yellow. Examples of quality markers are labeled.

B Volcano plot comparing the serum proteomes of COVID-19 patients at the time point of the highest Roche S-Ab test response and PCR-negative controls according to Fig 2B. Examples of quality markers are labeled.

C Volcano plot of a one-sample *t*-test comparing the sample at day 0 and the time point with the highest antibody levels according to Fig 3B. Examples of quality markers are labeled.

© 2021 The Authors

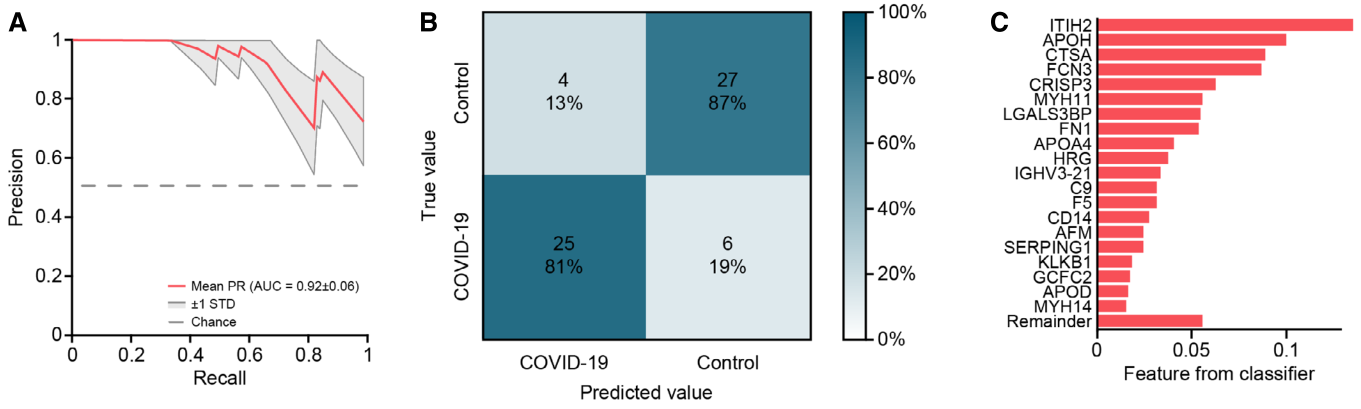

Figure EV3. Prediction whether a sample is from a COVID-19 positive patient or a PCR-negative control.

A The mean Precision-Recall (PR) curve (red) and PR curves ± 1 standard deviations are displayed (gray).
B Confusion matrix depicting the predicted and actual disease group of patients.
C Feature importance (weights) from the ML classifier averaged over all cross-validation runs.

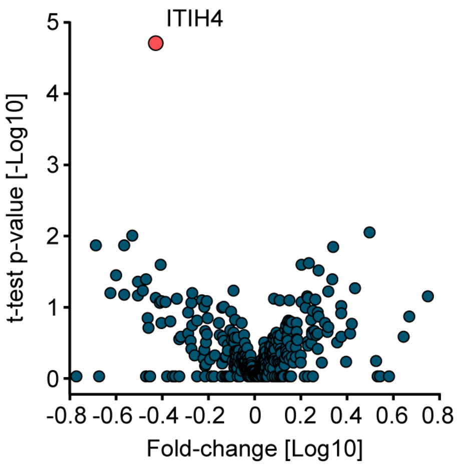

Figure EV4. Volcano plot showing the results of the comparison of 25 patients that survived COVID-19 infection and six patients that did not.

ITIH4 (red) was the only protein with a statistically significant difference.

 © 2021 The Authors

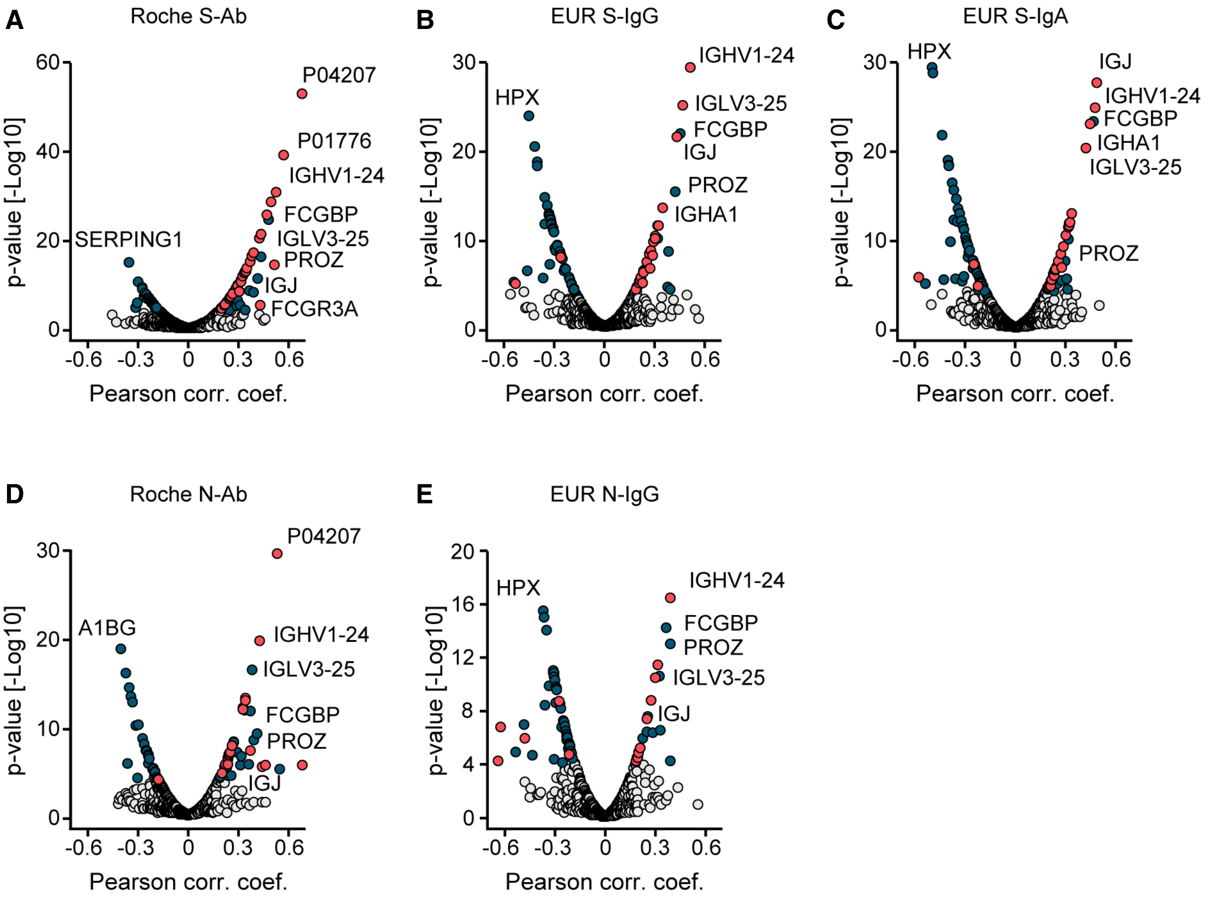

Figure EV5. Protein correlations to SARS-CoV-2 antibody assays.

A–E Correlation analyses of proteins with Roche S-Ab (A), EUR S-IgG (B), EUR S-IgA (C), Roche N-Ab (D), and EUR N-IgG tests (E). Antibodies are highlighted in red. Other significant proteins are highlighted in blue. Pearson correlation coefficients and *P*-values were calculated, and statistically significantly correlating proteins were determined using a Benjamini-Hochberg FDR correction.
